# Identifying Candidate Biomarkers of Ionizing Radiation in Human Pulmonary Microvascular Lumens Using Microfluidics—A Pilot Study

**DOI:** 10.3390/mi12080904

**Published:** 2021-07-29

**Authors:** Larry J. Millet, Richard J. Giannone, Michael S. Greenwood, Carmen M. Foster, Kathleen M. O’Neil, Alexander D. Braatz, Sandra M. Davern

**Affiliations:** 1 The Center for Environmental Biotechnology, The University of Tennessee, Knoxville, TN 37996, USA; lmillet1@utk.edu; 2 Bioanalytical Mass Spectrometry Group, Oak Ridge National Laboratory, Oak Ridge, TN 37831-6229, USA; giannonerj@ornl.gov; 3 Advanced Reactor Systems Group, Oak Ridge National Laboratory, Oak Ridge, TN 37831-6229, USA; greenwoodms@ornl.gov; 4 Molecular and Cellular Imaging Group, Oak Ridge National Laboratory, Oak Ridge, TN 37831-6229, USA; fostercm1@ornl.gov; 5 Oak Ridge Associated Universities, Oak Ridge, TN 37830, USA; oneilk@vt.edu; 6 Nuclear Energy and Fuel Cycle Division, Oak Ridge National Laboratory, Oak Ridge, TN 37831-6229, USA; braatzad@ornl.gov; 7 Radioisotope Science and Technology Division, Oak Ridge National Laboratory, Oak Ridge, TN 37831-6229, USA

**Keywords:** microfluidics, microvascular cells, γ radiation, proteomics, mass spectrometry

## Abstract

The microvasculature system is critical for the delivery and removal of key nutrients and waste products and is significantly damaged by ionizing radiation. Single-cell capillaries and microvasculature structures are the primary cause of circulatory dysfunction, one that results in morbidities leading to progressive tissue and organ failure and premature death. Identifying tissue-specific biomarkers that are predictive of the extent of tissue and organ damage will aid in developing medical countermeasures for treating individuals exposed to ionizing radiation. In this pilot study, we developed and tested a 17 µL human-derived microvascular microfluidic lumen for identifying candidate biomarkers of ionizing radiation exposure. Through mass-spectrometry-based proteomics, we detected 35 proteins that may be candidate early biomarkers of ionizing radiation exposure. This pilot study demonstrates the feasibility of using humanized microfluidic and organ-on-a-chip systems for biomarker discovery studies. A more elaborate study of sufficient statistical power is needed to identify candidate biomarkers and test medical countermeasures of ionizing radiation.

## 1. Introduction

Insecurities inherent to the global proliferation of radioactive materials demand that innovative medical countermeasures be identified to improve the response to radiological release, exposure, and contamination. Medical countermeasures have the potential to ameliorate the negative health effects of acute radiation syndrome, the delayed effects of acute radiation exposure, and multiple organ dysfunction syndrome that could result from radiation exposure.

Longitudinal epidemiological studies of radiological accidents identify that “… the symptomatology of organ system involvement could be traced not only to the pathophysiology of the rapidly turning over cell renewal systems but—of equal or more importance—to the vascular system and specifically, to the endothelial components” [1]. Degeneration and failure of the vascular system are primary contributors to dysfunction and failure of the body’s many organs, resulting in acute radiation syndrome, delayed effects of acute radiation exposure, and multiple organ dysfunction syndrome [2,3,4].

Chemical signals are produced and received by the single-cell-thick vascular endothelium that lines all blood vessels—signals that orchestrate blood vessel dilation, contraction, communication, and proliferative growth in tissues and organs of the body [5]. Following radiological insult, the vascular endothelium becomes the source and target of inflammation, which initiates a cascade of structural and functional breakdown of the vasculature. Capillary collapse and scarring result in a rigid ineffective circulatory system incapable of regeneration [6]. Nevertheless, a lack of mechanistic knowledge remains on the human vascular pathophysiology of radiation-induced injuries and an immune response to irradiated endothelial cells. Specifically, the identification of molecular pathways and targets is needed to develop medical countermeasure strategies that alleviate, stop, or reverse progressive vascular inflammation and fibrosis [7,8]. More concerted efforts are needed to identify the tissue-specific biomarkers that are predictive of the extent of tissue and organ damage and to identify medical countermeasures for treating individuals exposed to ionizing radiation [9,10]. The identification of biomarkers of radiation exposure has the potential to allow the resolving of pathobiological mechanisms of radiation-induced endothelial injury and permit the testing of effectiveness of candidate emergency medical countermeasures. With a greater knowledge of biomarkers of radiation exposure, early intervention may allow for natural cell recovery, or regeneration, to repair vascular damage [11,12,13,14]. However, the mechanisms of cellular response to gamma (γ) irradiation insults are not well understood, thus hampering efforts to design, select, or test candidate countermeasures.

Microfluidics enable the development and maintenance of a wide variety of organ-on-a-chip systems that constrain chemical volumes by limiting physical dimensions of cellular spaces in realms that are more in vivo-like than bulk dish or flask cultures [15,16,17]. Spatial-temporal chemical dynamics of cellular systems can easily be achieved at more realistic and relevant scales using microfluidics [18]. These miniature systems also minimize analyte dilution, thus allowing for improved detection of cell-released factors [19,20,21,22,23].

A vast number of microfluidic platforms have been created and implemented for vascular biology and bioengineering applications [24,25]. The bulk of these applications use co-culture constructs of multiple cell types toward the support of functional tissues [26,27,28]. Components involved in hydrogel formation, and fibroblast and microvascular endothelial cell seeding have been parameterized in microfluidics to instruct the development of prescribed microvascular network morphologies [29] for tissue specific engineering applications [30], including wound closure [31] and microtumor models [16,32], and for supporting the growth and maintenance of cellular spheroids [33].

Mass spectrometry of single cells has been achieved, but these methods are often destructive and only allow for a “snapshot” in time [34,35,36]. To our knowledge, no work to date demonstrates the ability to obtain a temporal resolution of early candidate biomarkers from direct radiation exposure of human cells or tissue in human organ or tissue systems using microfluidics.

In this work, we have established a 17 µL vascular lumen-on-a-chip platform for sustained cultures of human lung microvascular cells (HMVEC-L) in a monolayer under flow. This platform allows for the exposure of microvascular lumens to γ radiation for the temporal collection of cellular secretions through luminal perfusates and subsequent biomarker analysis. In this study, we generate proof-of-concept data using mass-spectrometry-based proteomics to analyze radiation damage. These results indicate that the detection of candidate biomarkers for radiation exposure can be obtained through this microfluidic platform. These microfluidics systems can be enhanced, augmented, and applied to this biological model or other human organ tissue structures to allow for further studies on irradiated cellular systems, neutrophil migration and immune modulation, γ-ionizing radiation induced fibrosis, and radiation-exposure metrics (phosphorylation of the histone protein H2AX (γ-H2AX)) of irradiated human-derived organs, in vitro.

## 2. Materials and Methods

### 2.1. Microfluidics

Chrome masks and microfluidics masters were produced in-house at the Center for Nanophase Material Sciences through an approved, peer-reviewed user project; microfluidic replicates were generated and assembled within the bio-affiliate laboratory at Oak Ridge National Laboratory. A laminar flow computational fluid dynamic model was performed with COMSOL Multiphysics 5.4 (Build: 346) utilizing the computer-aided design (CAD) import module for geometry uniformity between modeling and application. Single-layer microfluidics were fabricated from CAD drawings according to similar methods detailed previously [37]. Briefly, wafers were patterned with SU-8 2015 and developed with an SU-8 developer. Patterned wafers were etched with a modified Bosch process to produce raised structures (130 μm) for molding microfluidic channels. Wafers were coated with a trichloro(1H,1H,2H,2H-perfluoro-n-octyl)silane coating to facilitate replicate molding. Flexible attachment ports of silastic tubing (Helix Medical, Carpinteria, CA, USA) were molded in place during the polydimethylsiloxane (PDMS) pour-and-cure process using Duco cement (part #62435, Devcon, Hartford, CT, USA). PDMS replicas with integrated tubing were removed from the wafer, trimmed, cleaned (using 3M Scotch Magic tape, 3M, Maplewood, MN, USA), plasma treated, and baked to produce a single-layer PDMS microfluidic channel covalently attached onto a 25 mm × 75 mm microscope slide.

To prepare for cell culture studies, microfluidic systems were created, with care taken to prevent particulate contaminations. Microfluidics were sterilized under ultraviolet (UV) lights (2–3 min each side, Stratagene UV Stratalinker 2400). The channels were prepared for fluidic priming using a vacuum chamber to degas PDMS. The channels and glass were coated with poly-L-lysine (100 µg/mL water, sterile filtered), and rinsed with culture media to prevent the introduction of air before the direct seeding with HMVEC-L cells.

### 2.2. Cell Culture

HMVEC-L cells were procured (Lonza, Walkersville, MD, USA) and grown on poly-L-lysine coated microfluidics. Each microvascular microfluidic slide was retained in a 60 mm petri dish with sterile nanopure water to prevent dehydration during culture. T-75 flasks containing HMVEC-L cells were maintained in complete VascuLife media (Lifeline Cell Technologies, Frederick, MD, USA) with antibiotics included as supplements in VascuLife media kits. When the flask reached 80% confluence, cells were split using trypsin and VascuLife media. HMVEC-L cells were pelleted, rinsed, resuspended in 30 µL, perfused through the channels, and allowed to attach for 1 h before starting syringe pump for continuous perfusion at 0.5 µL/min. HMVEC-L cells in microfluidics were maintained up to 12 days in vitro. Immediately before γ irradiation, perfusion was stopped and the syringe with microfluidic system transported to the radiation source in a 37 °C, temperature-stable organ transport system (MT4-ET, B Medical Systems, Noblesville, IN, USA). After irradiation, cells were returned to the incubator and perfused with VascuLife media at 0.5 µL/min. Media were collected every 65 min to provide 30 µL media sample process volume for mass spectrometry; samples were immediately stored in microfuge tubes at −80 °C until they were processed for mass spectrometry. Stocks of untreated HMVEC-L cells were maintained in cryopreservation in VascuLife media supplemented with 10% fetal bovine serum and 10% dimethyl sulfoxide.

### 2.3. Gamma Irradiation Treatment

Confluent microvascular cultures (500–700 cells/mm^2^) were transferred to the Cobalt-60 (Co-60) γ radiation source within 60 mm petri dishes held inside a thermostable organ transport chamber maintained at about 37 °C. Independent to microfluidic sample processing, bulk microvascular cell cultures were calibrated with exposure to Co-60 γ ray dosages of 1, 5, 10, and 20 Gy. For proof-of-concept in this work, we exposed cells to 10 Gy Co-60 γ ray for γ-H2AX labeling and proteomic measurements; 10 Gy is a center value in the range of irradiation exposures, as discussed below. During microfluidic sample exposure to γ rays or mock treatment (approximately 5–7 min), microfluidic cell systems were kept under similar room-temperature conditions; both treated and mock-treated devices were immediately placed in the prewarmed transport chamber and returned to the cell culture incubator for perfusion and media collection. Radiation damage was assessed by immunocytochemistry for the formation of double-stranded breaks in DNA, which produce phospho-histone H2A.X foci at serine residue 139 (γ-H2AX) foci. Immunocytochemistry of γ-H2AX following 10 Gy irradiated HMVEC-L in microfluidics was performed after 15 days in vitro.

### 2.4. Immunocytochemistry

Cells were fixed with 4% electron-microscopy-grade paraformaldehyde in phosphate-buffered saline (PBS) for 10–15 min, followed by a PBS rinse, then permeabilized with 0.25% Triton™ X-100 in PBS for 10 min. The blocking agent was MAXblock™ Blocking Medium (2 h, room temperature and overnight, 4 °C), followed by a 10 min wash in MAXwash™ washing medium (Active Motif Inc., Carlsbad, CA, USA). Anti-phospho-histone H2A.X (serine 139) primary antibody clone JBW301 (MilliporeSigma, Burlington, MA, USA) was diluted to 2 µg/mL. The secondary antibody, Anti-Mouse IgG (H+L), F(ab’)2 fragment CF™ 488A (MilliporeSigma) was diluted to 1 µg/mL. Both antibodies were diluted in MAXbind™ Staining Medium (Active Motif Inc.). The cells were incubated in 50 µL of primary antibody solution (2 h, room temperature). Three MAXwash rinses (5 min each) were performed after the primary antibody. Secondary antibody labeling (50 µL, 1 h, 37 °C) was followed by four washes with MAXwash washing medium (5 min each). Rhodamine phalloidin was used to label the actin cytoskeleton (30 min, room temperature, 0.0165 µM in PBS) (Biotium Inc., Fremont, CA, USA). Cells were triple rinsed before 4′,6-diamidino-2-phenylindole (DAPI) (0.02 µg/mL PBS) labeling (VWR International Ltd., Lutterworth, Leicestershire, England). For data acquisition, labeled cells were triple rinsed and covered with 20 µL of Mowiol 4–88 (MilliporeSigma) solution per the manufacturer’s instructions (2008 Data sheet 777 Mowiol 4–88, Polysciences Inc., Warrington, PA, USA). The γ-H2AX imaging was performed with a Biotek Cytation-1 Cell Imaging Multi-Mode Reader (BioTek Instruments Inc., Winooski, VT, USA) and Gen5 Microplate Reader and Imager Software (catalog no. GEN5, BioTek Instruments Inc., Winooski, VT, USA).

### 2.5. Mass Spectrometry

HMVEC-L microvascular perfusates (30 µL) were stored at −80 °C until processed to obtain a biomarker library. The 30 µL samples of γ-irradiated and nonirradiated microvasculature microfluidics were prepared for liquid chromatography–tandem mass spectrometry (LC-MS/MS). Samples were diluted to 200 µL with denaturation/reduction buffer (4% sodium deoxycholate, 100 mM ammonium bicarbonate, 10 mM dithiothreitol), heated to 85 °C for 10 min, and treated with iodoacetamide (adjusted to 30 mM, 15 min at room temperature in the dark) to block disulfide-forming cysteine residues. Samples were passed through a 50 kDa molecular weight cut-off (MWCO) spin filter (Vivaspin 500; Sartorius, Gottingen, Germany) to remove serum albumin and other large proteins. Filter flow through was then transferred to a 10 kDa MWCO spin filter for on-filter proteolytic digestion with 1 µg proteomics-grade trypsin (Pierce) at 37 °C overnight, followed by a second addition of trypsin the following day for 3 h. The samples were then centrifuged at 12,500× *g* for 10 min to collect tryptic peptides that pass through the 10 kDa MWCO filter. The recovered peptides were then acidified to 0.5% formic acid, and the sodium deoxycholate precipitate removed with ethyl acetate as previously described [38]. Tryptic peptides were then autosampled onto an in-house-constructed, tri-phasic, 2D back column and desalted/cleaned up with a Vanquish uHPLC plumbed to a Q Exactive Plus high-resolution mass spectrometer (Thermo Fisher Scientific, Waltham, MA, USA) outfitted with a nanospray source as previously described [38]. Once desalted, peptides were then transferred via salt cut (500 mM ammonium acetate) to an in-house-pulled nanospray emitter (75 µm inner diameter fused silica packed with 30 cm of Kinetex C18 resin (5 micron particle size; Phenomenex, Torrance, CA, USA)) and analyzed by LC-MS/MS over a 120 min reversed-phase gradient as previously detailed [38].

### 2.6. Data Analysis

MS/MS data were then searched against the human proteome database (June 2017-build; UniProt) concatenated with common protein contaminants, and peptide spectral matches were scored, filtered, and assembled to proteins using the Proteome Discoverer software suite (Thermo Scientific; search algorithm: MS Amanda v.2; peptide scorer: IMP-Elutator) with the following parameters: MS1 tolerance ≤ 5 ppm, MS2 tolerance ≤ 0.02 Da, fully tryptic with 2 miscleavages, static modification on Cys = 57.0215 Da, dynamic modification on Met = 15.9949 Da. Peptide- and peptide-spectrum-match-level false discovery rates were assessed by hits to a reversed database and maintained at ≤0.01. High-confident peptide matches were then quantified by area under the curve and assembled to their respective proteins, whereby protein abundance was calculated by the summed abundance of all constituent peptides. Protein abundances were then log2-transformed and the resulting abundance distributions normalized and standardized by locally estimated scatterplot smoothing, median absolute deviation, and median centering via InfernoRDN [39]. Normalized protein abundances were then moved to Perseus proteome informatics software [40,41] to impute missing values and assess differences between irradiated and nonirradiated microvasculature microfluidic chips.

## 3. Results

**Figure 1:**
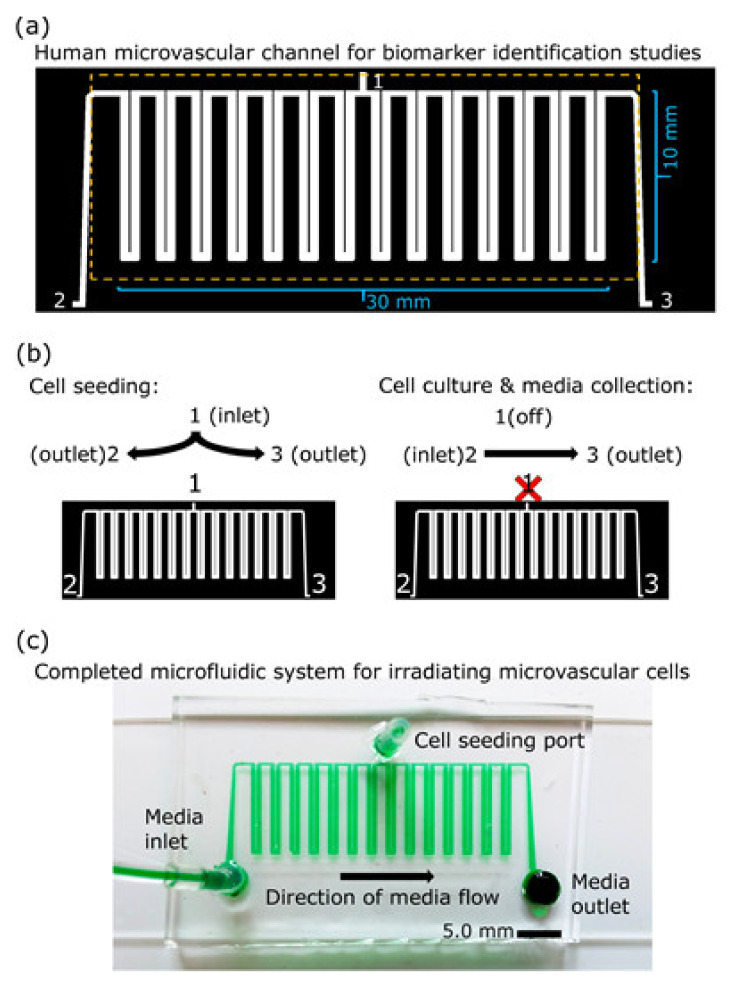
A large-scale microfluidic platform for generating microvascular lumens for biomarker analyses. (**a**) A schematic depicting the microfluidic platform design to maintain HMVEC-L cells and minimize dilution of analytes. The platform has three ports (1, 2, 3) that can be used for access points. Each of the 14 U-turns on the microfluidic chip contain 42 support posts (30 µm diameter) in channel dimensions of 21 mm (l), 550 μm (w), and 100 μm (h), giving 1.15 µL per U-turn; 14 U-turns and 15 interconnects (0.045 µL each) with a single inlet and outlet channel yields a total lumen volume of approximately 17 µL. Scale marks are shown (10 mm and 30 mm). (**b**) Diagrams for infusion flow direction for cell seeding and for culture maintenance are shown. Bilateral flow from center to end promotes uniform cell seeding throughout the long channel. During culture maintenance, the cell seeding port is sealed, and end-to-end perfusion flow maintains cell growth to achieve confluence of microvascular cells. (**c**) Image of a fabricated dye-filled microfluidic chip.

The initial platform design consisted of 15 U-turn segments in a single serpentine channel containing one inlet and one outlet. Upon seeding cells in the system, a continuum of cell-seeding densities settling throughout the length of the microfluidic channel was repeatedly observed. Confluence was obtained within the first four U-turn segments within a day of cell seeding, whereas the remainder of the chip progressed from high confluence to patchy then sparse cell adherence. The result of the initial platform was that most of the chip contained an insufficient number of cells that prevented a confluent microvascular lumen from forming. The second and final experimental platform used one fewer U-turn segment (total 14), and an additional cell-loading port situated at the center of the platform (Figure 1). Minor changes were also made to channel corners and barrier walls of the outlet. Seven U-turn segments were situated on each side of the central loading channel between the cell seeding port and the seeding outlet ports; the resulting microfluidic device contained a single 17 µL channel.

**Figure 2:**
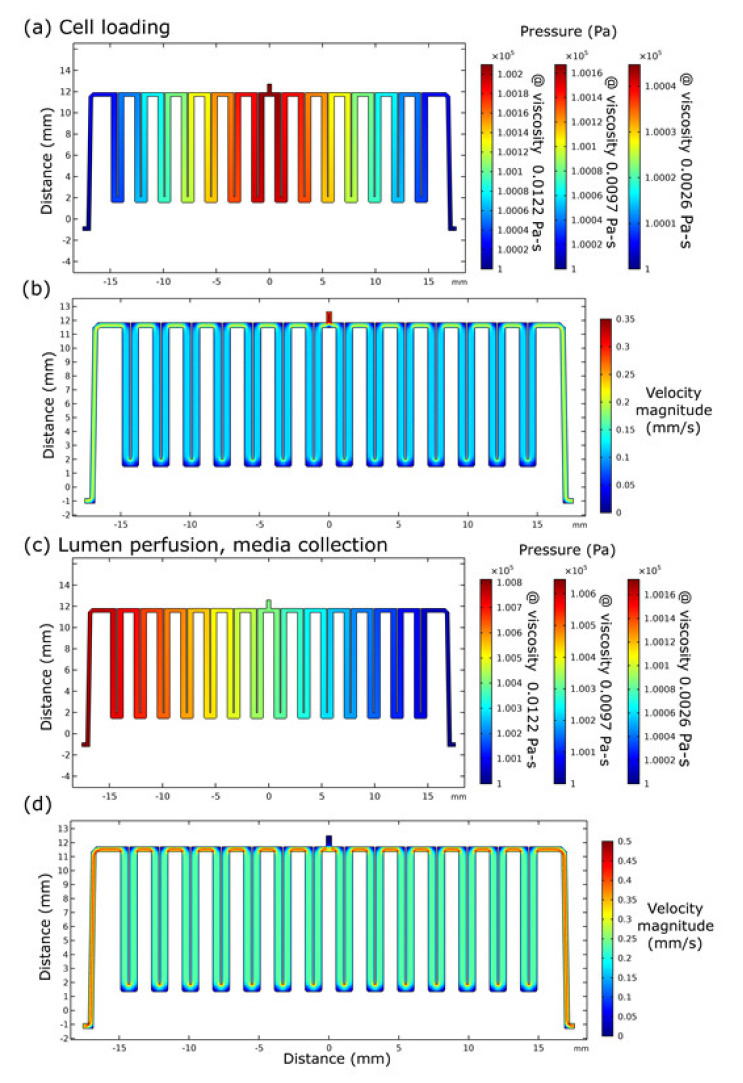
Computational fluid dynamics model velocity and pressure results in microfluidic channels for human microvascular endothelial lumens used for cell culture, irradiation, and media collection (**a**,**b**). During cell loading, a brief bidirectional flow introduces cells into the serpentine channel microenvironment under reduced velocity and pressure (**c**,**d**). End-to-end flow during lumen cell culture perfusion and media collection (0.5 µL/min) occurs with the middle cell loading port sealed; this perfusion system uses a slightly greater fluid velocity and pressure.

A computational fluid dynamics model was used to provide a preliminary pressure loss and flow behavior estimate in the experimental platform used for cell culture, ionizing radiation exposure, and sample collection. Cell loading occurs with a clean dislodged pellet of cells, with approximately 50% to 60% cells per unit volume. We modeled pressure drops in these microfluidic platforms using prior data for media heavily loaded with cells; published values for the apparent viscosities of cell solutions indicate that a 58% cell volume fraction has a viscosity of 0.0122 Pa-s (12.2 centipoise), a 52% cell volume fraction has an apparent viscosity of 0.0097 Pa-s (9.7 centipoise), and a 34% cell volume fraction has a viscosity of 0.0026 Pa-s (2.6 centipoise) [42]. The boundary conditions of the simulation were held constant: the inlet at a fixed, uniform volumetric flowrate of 0.5 µL/min at a density of 1000.0 kg/m^3^ and 1072.3 kg/m^3^ (for a range of media densities) and a fixed outlet pressure of 1 × 10^5^ Pa (atmospheric pressure, 1 ATM) [43]. For loading cells in the central portion of the channel, the modeling data (Figure 2) for the highest cell volume fraction (58%, viscosity 0.0122 Pa-s) show a negligible fluidic pressure drop of 0.002 Pa from the inlet to either outlet for bidirectional flow and 0.0004 Pa for the lowest cell volume fraction (34%, viscosity 0.0026 Pa) cited previously. The modeling data for loading cells in this platform through an end-to-end method show a pressure drop of 0.008 Pa for a 58% cell volume fraction (0.0122 Pa-s) and 0.0016 Pa for a 34% cell volume fraction (0.0026 Pa-s). Apart from channel corners, the modeling results also show that the flow velocity of cell loading through the central port is half (0.125–0.2 mm/s) that of the end-to-end loading (0.25–0.40 mm/s) (Figure 2) and that viscosity only affects pressure drop in these simulations, not flow velocity.

**Figure 3:**
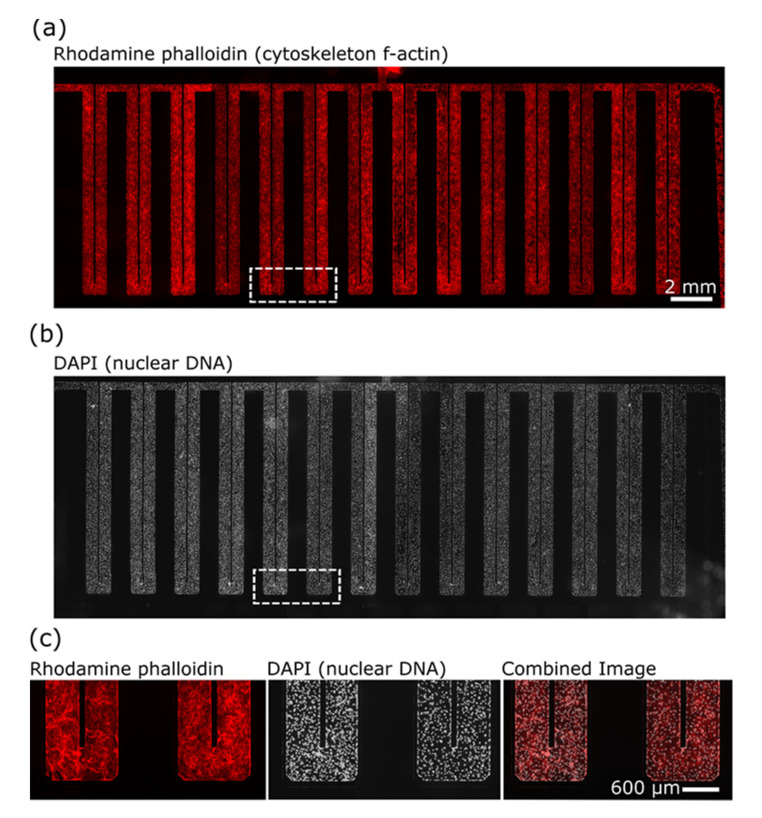
Human microvascular endothelial cells maintained in microfluidics for irradiation and biomarker identification. HMVEC-L cells were cultured with perfusion (0.25 to 0.5 μL/min) to confluence for 6–8 days to produce vascular lumens on a chip for irradiation and mass spectrometric analysis of perfusates. Composite images of the f-actin cytoskeleton, cellular nuclei, and merged images are shown here at 3 days in vitro. (**a**) Rhodamine-phalloidin-labeled f-actin cytoskeleton in HMVEC-L. (**b**) Cellular nuclei were labeled with DAPI. (**c**) Magnified end regions of microfluidic U-turn channels are identified by the dashed region of a–b.

HMVEC-L cells were cultured to confluence for 6–8 days to produce a vascular lumen (Figure 3). Lumen perfusion with physiologically relevant flow rates (0.5 μL/min, 0.1–0.2 mm/s, 10 dyne/cm^2^) maintained the HMVEC-L cells in microculture [44,45,46]. This was achieved based on successful perfusion values used in prior works and published values [47,48,49].

**Figure 4:**
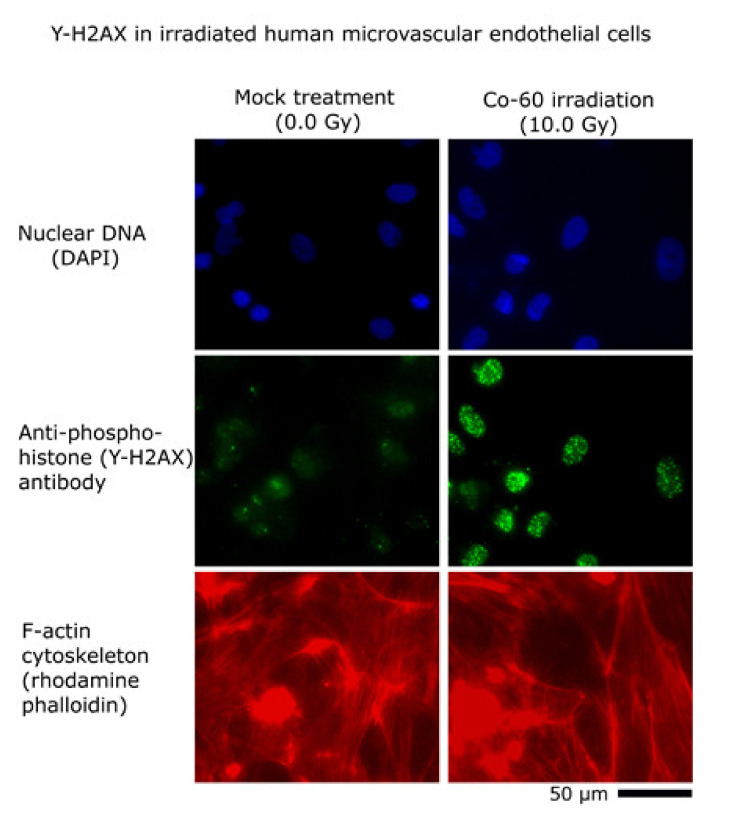
Irradiation of human microvascular endothelial cells with γ rays induces DNA damage. Images show DAPI-labeled cellular nuclei (top pair), γ-H2AX immunolabeling (middle pair), and rhodamine-phalloidin-labeled f-actin cytoskeleton (bottom pair). Double-stranded DNA breaks are evident in cells through immunocytochemistry for anti-phospho-histone H2A.X (serine 139), commonly termed γ-H2AX. Cells not exposed to γ rays (left image column) show very little γ-H2AX expression compared to cells exposed to 10 Gy of γ rays (right image column) from Co-60 source. The 50 µm scale bar applies to all six images.

Irradiation of human microvascular endothelial cells with γ rays induces double-stranded DNA breaks, damage that is evident in cells through immunocytochemistry for anti-phospho-histone H2A.X (serine 139) (γ-H2AX). Mock-treated cells (0.0 Gy, no γ irradiation) show few to no γ-H2AX foci per cell nuclei, whereas HMVEC-L cells exposed to 10 Gy of γ irradiation produce substantial γ-H2AX foci per nuclei at 3 h postexposure (Figure 4). The effect of ionizing radiation exposure and mock treatments to HMVEC-L cells was validated using γ-H2AX labeling and imaging.

**Figure 5:**
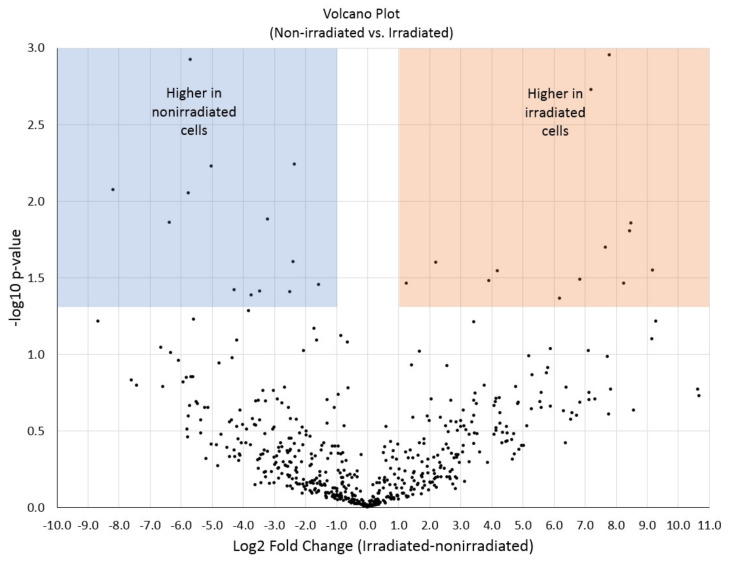
Volcano plot summarizing the proteomic results obtained from microvascular microfluidic lumen samples exposed to Co-60 γ radiation versus nonirradiated controls. Ultra-performance liquid chromatography (UPLC)-driven nanoelectrospray high-resolution tandem mass spectrometry and human proteome database analysis derived 26 statistically significant proteins that change in abundance between irradiated and nonirradiated tissue chip platforms (blue and orange boxed regions).

Preliminary proteomic inquiry of a few samples (*n* = 3, irradiated; *n* = 2 nonirradiated) with temporally collected luminal flow through both irradiated and nonirradiated cells identified a total of 568 proteins in aggregate, 176 of which were found in all samples and conditions. Sixty-three proteins were exclusive to irradiated cells. To garner a more quantitative perspective, proteins were log2-transformed, normalized, and compared across conditions. High-abundance differences were assessed by *t*-test, and results were depicted as a volcano plot (Figure 5). Using a *p*-value cutoff of 0.05 (-log10 *p*-value = 1.301; *y*-axis) and a fold-change cutoff of one log2 unit (minimum 2× difference), 26 proteins were found to exhibit differential abundance, with 13 higher in irradiated cells and 13 higher in nonirradiated cells. Several interesting candidate proteins linked to cell growth and differentiation, cancer progression and prognosis, and other oncogenetic processes were found to be more abundant in irradiated cells. From these data, a preliminary summary of candidate protein biomarkers was obtained (Figure 5) and correlated to γ irradiation exposure. Through replication, randomization, and blinded sampling, we obtained 35 statistically significant candidate compounds that are different between γ irradiation exposure and mock controls. Protein abundance was derived at the peptide-level (MS1) by measuring chromatographic area under the curve and then normalizing. The results were compared across replicates (*n* = 3) and conditions with significant differences assessed by *t*-test and ANOVA using Fisher’s least significant difference post hoc correction or Benjamini and Hochberg false discovery rate methods [50,51].

## 4. Discussion

Microfluidic technologies afford the ability to improve the chain of custody for cells and cellular solutions. Microfluidics combined with analytical chemistry have the potential to resolve the identities of cell-derived compounds needed for advancing biosystems research, producing medicinal compounds, and for advancing radiation biology [37,52,53]. The fluidic modeling data for this system were informative for guiding the perfusion of the microfluidic microvascular lumen. However, modeling does not fully capture the degree of challenge and complexity for loading cells in solution into a channel—essentially a flowing suspension of sticky semibuoyant spheres passing through a spatially restricted domain. Other researchers have modified the specific gravity of culture media formulations in an effort to adjust the maintenance frequency of sensitive cell populations [43]. The first channel built consisted of a long serpentine channel containing a single inlet and a single outlet. This two-port channel proved difficult for loading cells because they would adhere in an uneven distribution, progressing from high cell confluence to sparse seeding throughout the channel before clogging the fluidic channel. The resulting nonuniformity was unrecoverable, even if seeded from both directions, which became a barrier to throughput and platform success.

In the second version of the channel, we incorporated a loading port midway across the length of the channel, thus enabling facile loading of cells. After the cell loading and attachment, the port was sealed for end-to-end perfusion (Figure 1). Loading cells in microfluidics can be challenging. For example, cells can quickly attach one to another to form clusters that are too large for the channel and thus impede flow, a phenomenon not captured or described by these simulation data (Figure 2). Clumping and clogging can happen more readily in smaller-sized, low-volume channels, but the low cell density per unit volume can easily be overcome by cell division if the cells are mitotic and have low contact inhibition. As cells achieved and maintained confluence within our designed microfluidic device, the microvascular lumen they formed was then ready for irradiation, perfusion, and media collection.

The prior implementation of microfluidic systems for cell-based mass spectrometry has informed the process flow optimization for identifying analytes from cellular populations for advanced chemical analyses in peptidomics, metabolomics, and proteomics [21]. For example, microfluidic-assisted mass spectrometry has benefited neuroscience for the identification and label-free quantification of peptides released from neurons—fragile, intricate cells with high surface-to-area volume ratios [23,54,55]. Notwithstanding the successes amalgamating microfluidics with mass spectrometry, the pairing of these techniques is still in an optimization phase [22]. Microfluidics have been used for biodosimetry studies (quantitative nuclease protection assay) for high-throughput triage scenarios [56], γ-H2AX immunofluorescence cytometry [57,58], and gene expression assays of blood cells [59]. Recent reports indicate that more should be done to apply microfluidic radiobioassays to understand cellular physiology and pharmacokinetics [60].

In this pilot project, we chose to work with monocultures of primary human microvascular endothelial cells in microfluidics to obtain feasibility data for biomarker discovery studies of γ-irradiated cells. Using a 17 µL serpentine channel (Figure 1 and Figure 2) enables adequate perfusion and serial collection of samples across time for mass spectrometry. Our use of a homotypic, confluent monolayer of cells structured in a vascular lumen is foundational for subsequent studies that use multiple cellular populations comprising more complex organ-on-a-chip tissue forms. Resolving chemical signatures of individual cell types is also a prerequisite for irradiation studies of heterogenous tissues, where the reconciliation of proteomic results may involve the deconvolving of unique, synergistic, tissue-derived identities. A tissue construct may provide more meaningful and unique signals that come from paracrine signaling between multiple cell types. Summating results from multiple homotypic samples may not provide the same level of proteomic complexity as would a heterogenous tissue. In this device, vascular cells arrive at confluence (Figure 3) before initiating γ ray exposure (Figure 4); they do so in a microfluidic volume with structure and operation to support serial sample collections for subsequent mass spectrometry. Our results show that this microfluidic domain has enough cellular content, media exposure, and process volume to provide sufficient sample quantity to identify early candidate biomarkers of radiation (Figure 5) in monolayers and in organ-on-a-chip tissue constructs. These results are encouraging for subsequent studies to identify early biomarkers of radiation injury.

To our knowledge, omics-level studies of human-derived cells or tissue structures using microfluidics and ionizing radiation have not been performed; rather, subject-limited applications with microfluidics have been achieved [53]. To this end, we performed this pilot feasibility study as a proof of principle for the microfluidic-based approach to radiobiological studies. Pilot studies should first confirm an innovation to be feasible and achieve or clarify certain design aspects but need not prove the effectiveness of the study. In this work, we have identified design rules and made modifications to procedural requirements that enable microfluidics to achieve a large enough microvascular lumen volume to enable reproducible mass spectrometry of the microvascular perfusate. This pilot study paves the way for a large-scale project of sufficient statistical power to enable further testing and validation—a study where a list of candidate biomarker identities that can be tested with medically relevant countermeasures are identified.

Two medical countermeasure drug application experiments have been performed by other researchers using human gut-on-a-chip and bone marrow-on-a-chip models [61,62]. In a separate study, global protein expression of neurovascular units and uncoupled blood brain barriers developed in organ-on-a-chip systems were analytically compared to identify novel expression changes and unpredicted identities of metabolites that maintain neuronal functionality [63]. Recently, we used microfluidics to achieve label-free, time-resolved exometabolite sampling of growing plant root exudates through nanoporous interfaces. The results showed that microfluidic sampling methods enabled tracking the distribution of sucrose-derivative products from the proximal and distal microenvironment and the exudate identification using extracted ion chromatography [19].

Our preliminary analyses focused on measuring differential abundances of secreted proteins across irradiated and nonirradiated cells. For this feasibility study, we chose to use 10 Gy irradiation dose as a midpoint between conventional lower dose radiotherapy (1–3 Gy) and higher dose radiosurgery (10 Gy to 35 Gy) [64,65]. High dose treatments are used in single dose fractions for large brain metastases and other focal lesions such as small peripheral lung tumors. Flash therapy administers higher doses of 10–20 Gy at a higher rate of delivery [66,67]. Therefore, evaluating the effect of higher radiation dose on biomarker release is important to consider for patients receiving radiotherapy. It is also an exposure to be considered for nuclear radiation events, such as a deliberate radiological release, e.g., a “dirty bomb”.

Proteins of the microvasculature lumen were analyzed by high-resolution LC-MS/MS. The resulting peptide fragmentation data were searched and assigned peptides to their respective proteins, then contrasted across irradiated and nonirradiated microvasculature lumens to identify potential protein biomarkers of γ radiation exposure. Advances in bottom-up proteomic sample preparation methodologies enable microliter-scale proteomic measurements in minimal microliter-scale sample volumes [68]. Coupled with UPLC-driven nanoelectrospray high-resolution tandem mass spectrometry and microscale peptide fractionation, several hundreds to thousands of proteins can be reproducibly identified and quantified [69]. In this pilot study, we identified upward of 500 human proteins from the microvasculature flow through. Of these, 13 were found to be reproducibly increased in abundance upon ionizing radiation exposure. Encouragingly, several biomarker candidates related to cancer progression and/or prognosis have been found; however, pilot studies with low statistical power are not suitable for sequencing biomarkers. Experimental replicates greater than *n* = 3 are required before reporting statistically significant chemical identities. Nevertheless, these results are encouraging and lend credence to the applicability of microfluidic endothelial systems and organ-on-a-chip systems for biomarker discovery studies.

## 5. Conclusions

The few replicates of this pilot study demonstrate the feasibility of using humanized microfluidic and organ-on-a-chip systems for biomarker discovery studies. A more elaborate study of sufficient statistical power is needed to identify candidate biomarkers and test medical countermeasures of ionizing radiation to mitigate the resulting inflammatory and vascular disease. Obtaining these results with microfluidic systems is encouraging for further biomarker analysis studies and for implementing complex tissue architectures available through organ-on-a-chip systems. The primary benefit of these miniature systems is the ability to establish complex human tissue architecture for irradiation while minimizing analyte dilution. These microfluidic platforms easily enable spatiotemporal sampling—minutes to hours or even days—of the fluidic microenvironment off the same sample, thus eliminating the need for many multiples of parallel snapshots that are often required from bulk cultures. An added advantage of this approach is that conventional biochemical assays are moving to smaller analyte volumes through automated systems, thus allowing for improved detection of cell-released factors.
